# Increased juvenile predation is not associated with evolved differences in adult brain size in Trinidadian killifish (*Rivulus hartii*)

**DOI:** 10.1002/ece3.2668

**Published:** 2017-01-12

**Authors:** Shannon M. Beston, Whitnee Broyles, Matthew R. Walsh

**Affiliations:** ^1^ Department of BiologyUniversity of Texas at ArlingtonArlingtonTXUSA

**Keywords:** brain size evolution, early‐life mortality, extrinsic mortality, juvenile mortality, life‐history evolution, predator–prey interactions

## Abstract

Vertebrates exhibit extensive variation in brain size. The long‐standing assumption is that this variation is driven by ecologically mediated selection. Recent work has shown that an increase in predator‐induced mortality is associated with evolved increases and decreases in brain size. Thus, the manner in which predators induce shifts in brain size remains unclear. Increased predation early in life is a key driver of many adult traits, including life‐history and behavioral traits. Such results foreshadow a connection between age‐specific mortality and selection on adult brain size. Trinidadian killifish, *Rivulus hartii,* are found in sites with and without guppies, *Poecilia reticulata*. The densities of *Rivulus* drop dramatically in sites with guppies because guppies prey upon juvenile *Rivulus*. Previous work has shown that guppy predation is associated with the evolution of adult life‐history traits in *Rivulus*. In this study, we compared second‐generation laboratory‐born *Rivulus* from sites with and without guppies for differences in brain size and associated trade‐offs between brain size and other components of fitness. Despite the large amount of existing research on the importance of early‐life events on the evolution of adult traits, and the role of predation on both behavior and brain size, we did not find an association between the presence of guppies and evolutionary shifts in *Rivulus* brain size. Such results argue that increased rates of juvenile mortality may not alter selection on adult brain size.

## 1 Introduction

It has long been known that vertebrates exhibit extensive variation in brain size (Bauchot, Bauchot, Platel, & Ridet, 1977; Crile & Quiring, 1940; Jarvis et al., 2005; Mink, Blumenschine, & Adams, 1981; Striedter, 2005; Taylor & van Schaik, 2007). There are clear fitness benefits associated with a larger brain as brain size is positively correlated with increased intelligence, cognition, learning capability, population persistence, and decreased susceptibility to predation (Sol & Lefebvre, 2000; Tebbich & Bshary, 2004; Shultz & Dunbar, 2006a; Sol, Szekely, Liker, & Lefebvre, 2007; Sol, Bacher, Reader, & Lefebvre, 2008; Overington, Morand‐Ferron, Boogert, & Lefebvre, 2009; Barrickman, Bastian, Isler, & van Schaik, 2008; Amiel, Tingley, & Shine, 2011; Reader, Hager, & Laland, 2011; Kotrschal et al., 2013b; MacLean et al., 2014; Kotrschal et al., 2015a; Kotrschal, Corral‐Lopez, Amcoff, & Kolm, 2015b; Benson‐Amram, Dantzer, Stricker, Swanson, & Holekamp, 2016; but also see Drake, 2007). Key hypotheses, such as the expensive tissue hypothesis (i.e., expensive metabolic cost of brain tissue) (Aiello & Wheeler, 1995; Isler & van Schaik, 2009) and energy trade‐off hypothesis (increased encephalization leads to trade‐offs with other functions) (Isler & van Schaik, 2006a,b, 2009; Navarrete, van Schaik, & Isler, 2011; Tsuboi et al., 2015), recognize that brain tissue is costly and that fitness trade‐offs likely underlie increased encephalization (Aiello & Wheeler, 1995). Research has indeed shown that increased allocation to brain tissue leads to declines in other components of fitness (Kaufman, Hladik, & Pasquet, 2003; Kotrschal et al., 2013a; Mink et al., 1981; Navarrete et al., 2011; Raichle & Gusnard, 2002; Tsuboi et al., 2015). The observed costs and benefits of brain size, as well as the connections between brain size and fitness, foreshadow that variation in ecological factors have the potential to exert selection and influence observed patterns of brain size variation (Gittleman, 1994). Yet, the specific ecological drivers of brain size variation have largely remained elusive.

Recent work has identified a role for predation as an important selective force on the evolution of vertebrate brain size (van der Bijl, Thyselius, Kotrschal, & Kolm, 2015; Burns & Rodd, 2008; Edmunds, Laberge, & McCann, 2016; Kotrschal et al., 2015a; Shultz & Dunbar, 2006a; Walsh, Broyles, Beston, & Munch, 2016). Selection for larger brains in captive populations of guppies is associated with increased cognitive function and declines in susceptibility to predation (Kotrschal et al., 2013a,b, 2015b). However, work on natural fish populations in other species yielded the opposite trajectory of evolution. Walsh et al. (2016) compared populations of Trinidadian killifish, *Rivulus hartii*, from sites that differ in the presence and absence of large predators for variation in brain size. This work showed that male, but not female, *Rivulus* from sites with large piscivores have evolved smaller brains when compared to fish from sites that lack predators. Such sex‐specific differences may be related to known differences in fish behavior and learning abilities between sites that differ strongly in predation intensity (Archard & Braithwaite, 2011; Benson‐Amram et al., 2016; van der Bijl et al., 2015; Brydges, Heathcote, & Braithwaite, 2008; Cousyn et al., 2001; DePasquale, Wagner, Archard, Ferguson, & Braithwaite, 2014; Dingemanse et al., 2007; Fraser, Gilliam, Daley, Le, & Skalski, 2001; Gilliam & Fraser, 2001; Harris, Ramnarine, Smith, & Pettersson, 2010; Hembre & Peterson, 2013; Kotrschal et al., 2013a,b; Lima & Dill, 1990; Plijanowska, Weider, & Lampert, 1993; Tulley & Huntingford, 1988; Urban, 2007). For example, fish that experience weak levels of predation are faster learners and have better spatial cognition than fish that are exposed to higher rates of predation (Brydges et al., 2008; DePasquale et al., 2014). Male *Rivulus* with larger brains are potentially favored in safer environments due to the fitness benefits that may result from better problem‐solving behavior and increased cognition (Walsh et al., 2016). The current literature clearly illustrates a connection between predation regime and brain size evolution, but given the inconsistent nature of the results from this growing body of work (van der Bijl et al., 2015; Burns & Rodd, 2008; Gonda, Herczeg, & Merila, 2009a,b, 2011; Kotrschal et al., 2015a; Shultz & Dunbar, 2006b; Walsh et al., 2016), the generality of such conclusions requires further testing.

It is well known that the trajectory of evolution for many classes of traits depends upon the age and/or size classes that experience predator‐induced mortality (Brown, 2003; Charlesworth, 1980; Jonsson & Jonsson, 2014; Sih, Kats, & Maurer, 2003; Urban, 2007). For example, increased rates of juvenile predation are associated with the evolution of delayed maturation and decreased reproductive effort (Reznick & Endler, 1982; Sparkes, 1996a,b; Walsh & Reznick, 2009; Wellborn, 1994). Similarly, juvenile exposure to predation can influence adult behavior and learning (Bell & Sih, 2007; Jonsson & Jonsson, 2014; Lonnstedt, McCormick, & Chivers, 2012; Sparkes, 1996b). For instance, Bell and Sih (2007) showed that predator exposure as juveniles induces increased aggressive behavior and boldness as adults. Links between juvenile mortality and shifts in adult traits, especially adult behavior and learning, imply that mortality early in life may be an important selective force on adult brain size.

In addition to sites with large predators, *Rivulus* are also found in localities where juveniles are the target of predation (Fraser, Gilliam, MacGowan, Arcaro, & Guillozet, 1999; Gilliam, Fraser, & Alkinskoo, 1993; Walsh, Fraser, Bassar, & Reznick, 2011). *Rivulus* are located in sites with guppies *Poecilia reticulata* (hereafter *Rivulus/*guppy “RG” sites), which are located tens of meters downstream from sites in which *Rivulus* are the only species present (hereafter *Rivulus‐*only “RO” sites). The abundances of *Rivulus* decline dramatically at the point of contact with guppies (2–3× decline) because field and laboratory experiments have shown that adult guppies prey upon juvenile *Rivulus* (Fraser & Lamphere, 2013; Furness & Reznick, 2014; Walsh et al., 2011). *Rivulus* quickly attain a size that exceeds the gape of guppies (Furness & Reznick, 2014). Thereafter, *Rivulus* exhibit significantly faster rates of individual growth in RG versus RO localities (Furness & Reznick, 2014; Walsh et al., 2011). This increase in growth likely reflects increased per capita food availability in RG sites, which is likely an indirect consequence of increased gape‐limited predation by guppies (Walsh et al., 2011). Previous work has shown that these direct (larval mortality) and indirect (increased food) effects of guppies are associated with local adaptation in the life‐history traits of *Rivulus* between RG and RO communities (Walsh & Reznick, 2009, 2010, 2011). Such work clearly shows that increased predation by guppies can exert selection on *Rivulus*. Therefore, these interactions between *Rivulus* and guppies provide a means to test how size‐structured interactions shape the evolution of brain size when predatory mortality is presumably limited to early developmental stages in prey.

Here, we tested for genetically based differences in brain size and associated trade‐offs between brain size and other components of fitness (i.e., development rate; gut size) between *Rivulus* from three RG and three RO sites. We compared *Rivulus* from RG and RO sites for differences in brain and gut size using existing specimens stemming from previous second‐generation common garden‐reared experiments (see Walsh & Reznick, 2010, 2011). This prior work evaluated the evolutionary consequences of the direct and indirect effects of guppies by rearing all populations on two food levels that match the known differences in growth. The specimens stemming from this work thus allow us to test the effects of the direct and indirect consequences of interactions with guppies on the evolution of brain size in *Rivulus*. If increased juvenile mortality alters selection on adult brain size, then we predict that we will observe a similar trajectory of evolution as driven by predators capable of consuming all size classes of prey (see Walsh et al., 2016) and that male *Rivulus* in RG sites will exhibit smaller brains than fish from corresponding RO communities. This is due to the potential fitness benefits associated with large brain size in nonrisky environments, as well as the benefits of allocating energy elsewhere in high‐predation environments (Shultz & Dunbar, 2006b). A failure to observe this pattern may indicate that mortality early in life does not alter adult behavior or learning capabilities and, by association, brain size.

## 2 Materials and Methods

The experimental methodology is previously published (Walsh & Reznick, 2010, 2011) and is summarized here. *Rivulus* were collected from RO and RG communities from the Aripo, Guanapo, and Quare rivers in January 2007. Twenty to 25 wild‐caught males and females were used to establish laboratory populations for the common garden experiments. These wild‐caught females and males from the same locality (i.e., same river and same community) were paired in a 9‐L tank. Over the course of approximately 20 days, eggs from each pair were harvested and reared in petri dishes. Once hatched, eight to 10 larvae were placed in a 9‐L aquarium and fed a diet of liver paste and brine shrimp *nauplii* ad libitum. Once sex was identifiable (~50 days post hatch), fish were placed in tanks of two to four fish with equal ratios of males to females until sexual maturation was reached.

The second common garden generation was generated using six to eight randomly paired killifish from each population (see Fig. S1 of Walsh & Reznick, 2010). We then collected eggs from all pairings for 10–20 days. Upon hatching, eight to 12 larvae were placed in 9‐L aquaria and were reared under the same conditions as the previous generation. After 20 days, eight fish from each pairing were randomly selected and individually placed in 9‐L tanks until maturation. These fish were randomly allocated to either (1) high‐food (HF) or (2) low‐food (LF) treatments. *Rivulus* exhibit a rate of growth that is two to three times faster in RG communities when compared to RO localities (Walsh et al., 2011). The high‐food levels used in these experiments thus sustained a growth rate that approximates that observed in the RG communities, while the low‐food treatments were designed to mimic growth rates in RO communities (Fraser et al., 1999; Walsh & Reznick, 2008). All fish were then reared until maturation. Males were euthanized at maturation, while females were euthanized following a 2‐week period of egg collection after maturation (Walsh & Reznick, 2010, 2011). Each day, all fish were euthanized and preserved in the morning (~16 hours after the prior afternoon feeding). Such timing allows for sufficient processing of food, eliminating potential bias that might be associated with varying amounts of food left in the gut upon preservation (Walsh et al., 2016). Specimens were preserved in 5% formalin for approximately 8 years prior to being dissected for brain and gut size beginning in August 2015.

### 2.1 Brain weight and gut size measurements

We dissected the brain from each specimen by cutting from the top of each gill slit to remove the lower jaw and any tissue between the mouth and braincase. Each brain was blotted dry prior to measuring the wet weight of the brain (mg). The gut was removed by first cutting from the tip of the anus to remove the posterior end. The fish was then cut where the esophagus meets the stomach. Each gut was blotted dry and measured for wet weight (mg).

### 2.2 Statistical design and analyses

The dependent variables included brain and gut size. All variables were analyzed using general linear models with fish community (*Rivulus‐*only, *Rivulus/*guppy), food treatment (high, low), river (Aripo, Guanapo, Quare), and sex (male, female) and all interactions included as fixed effects (SPSS v.23, IBM Corporation). Body weight was included as a covariate in all analyses. Brain size, gut weight, and total weight were ln transformed to better linearize the data.

### 2.3 Trait correlations

To explore trade‐offs between brain size and gut size, and age at maturation, Pearson correlations were performed between brain size versus age at maturation and gut size versus age at maturation between RO and RG sites for each river. The data for age at maturation have been previously published (Walsh & Reznick, 2010, 2011). To correct for body size, residuals from the general linear model were used with body size as a covariate.

## 3 Results

### 3.1 Fish community effects

**Figure 1:**
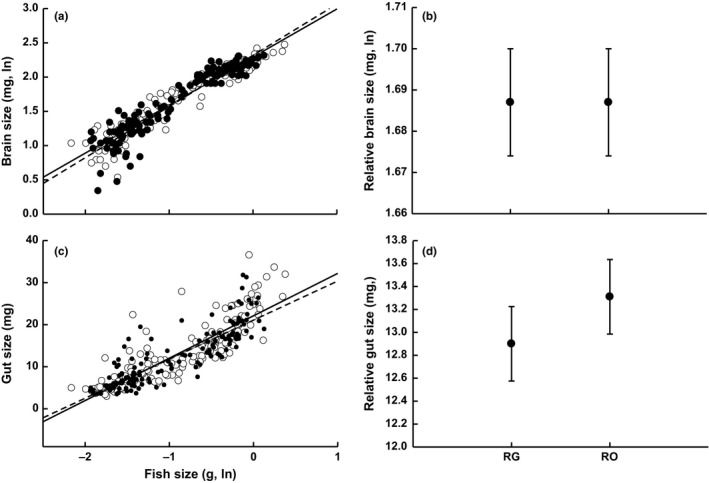
Variation in brain and gut size between fish communities. (a) fish size versus brain size, (b) relative brain size, (c) fish size versus gut size, (d) relative gut size. Panels a, c: closed circles, solid regression line—*Rivulus*‐only sites; open circles, dashed regression line—*Rivulus*/guppy sites. Panels b, d: RG = *Rivulus*/guppy, RO—*Rivulus*‐only. Differences in absolute and relative brain and gut were not significant (*p* > .05) between RG and RO sites. The data points for relative brain and gut size reflect the estimated marginal means at the mean of the covariate. Error = ±1 *SE*

**Figure 2:**
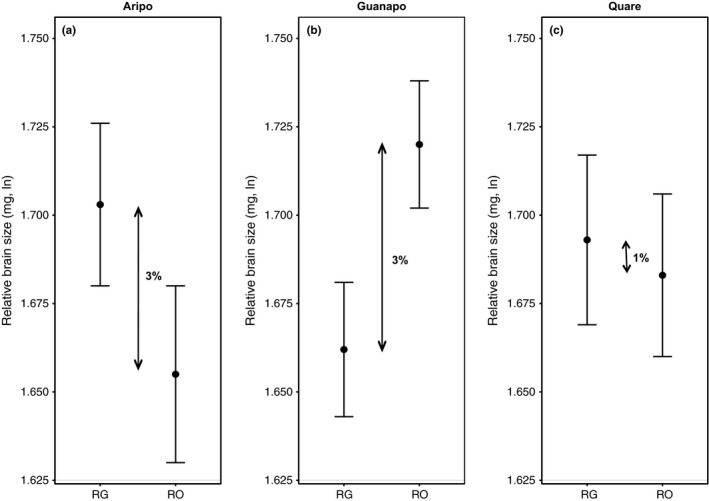
Brain size differences between fish communities depended upon river. Relative brain size of *Rivulus* from *Rivulus/*guppy (RG) and *Rivulus‐*only (RO) communities in the (a) Aripo, (b) Guanapo, and (c) Quare rivers

Differences in absolute and relative brain size and relative gut size were nonsignificant (*p* > .05) between RO and RG populations (Table 1; Figure 1). We observed marginally nonsignificant (*p* < .1) differences between RG and RO sites for absolute gut size (Table 1; Figure 1). *Rivulus* from RO sites exhibited a gut size that was 4% larger than RG sites (Figure 1). Differences in relative brain size between fish communities depended upon the river of origin (i.e., significant “river × population” interaction; Figure 2, Table 1). *Rivulus* from RG localities in the Aripo and Quare rivers exhibited a relative brain size that was 3% and 1% larger than *Rivulus* from RO sites, respectively (Figure 2). The opposite pattern of divergence was observed in fish from the Guanapo River, as the brain size of *Rivulus* from the RO site was 3% larger than the corresponding RG population (Figure 2). The “river × population” interaction was not significant for relative gut size (Table S1).

**Table 1:** Analyses of brain and gut size variation. Significant terms are indicated in bold

	*df*	Absolute brain size (mg)		Relative brain size (mg)		Absolute gut size (mg)		Relative gut weight (mg)	
		*F*	*p*	*F*	*p*	*F*	*p*	*F*	*p*
Covariates									
Fish size	1	**…..**	**…..**	**311.6**	**<.001**	**…..**	**…..**	**161**	**<.001**
Main effects									
Predation	1	2.43	.12	0	.99	3.61	.058	0.43	.51
Food	1	**21.95**	**<.001**	**5.57**	**.019**	**13.49**	**<.001**	3.71	.055
River	1	**15.96**	**<.001**	0.16	.85	**7.32**	**.001**	0.92	.4
Sex	1	**948.98**	**<.001**	2.16	.14	**400.18**	**<.001**	3.72	.055
Predation × Food	1	0.85	.36	0.24	.62	0.76	.39	0.022	.88
Predation × River	1	1.14	.32	**3.37**	**.036**	1.38	.25	0.25	.78
Predation × Sex	1	2.22	.14	0.24	.62	1.23	.27	0.033	.86
Food × River	1	1.95	.14	0.91	.4	1.17	.31	0.28	.76
Food × Sex	1	3.37	.068	**7.12**	**.008**	0.37	.54	0.74	.39
River × Sex	1	**5.75**	**.004**	2.07	.13	**10.57**	**<.001**	0.99	.37
Predation × Food × River	1	0.46	.63	0.74	.48	0.28	.76	0.59	.56
Predation × Food × Sex	1	0.01	.92	0.06	.8	0.64	.42	0.79	.38
Predation × River × Sex	1	0.54	.58	1.39	.22	0.57	.57	0.6	.55
Food × River × Sex	1	0.62	.54	0.83	.44	0.49	.61	0.47	.63
Predation × Food × River × Sex	1	1.02	.36	0.9	.41	0.12	.89	0.45	.64
Error *df*		261		260		265		264	

*F*,* F*‐values; *p*,* p*‐values; *df*, numerator degrees of freedom; Error *df*, denominator degrees of freedom.

### 3.2 Food effects

**Figure 3:**
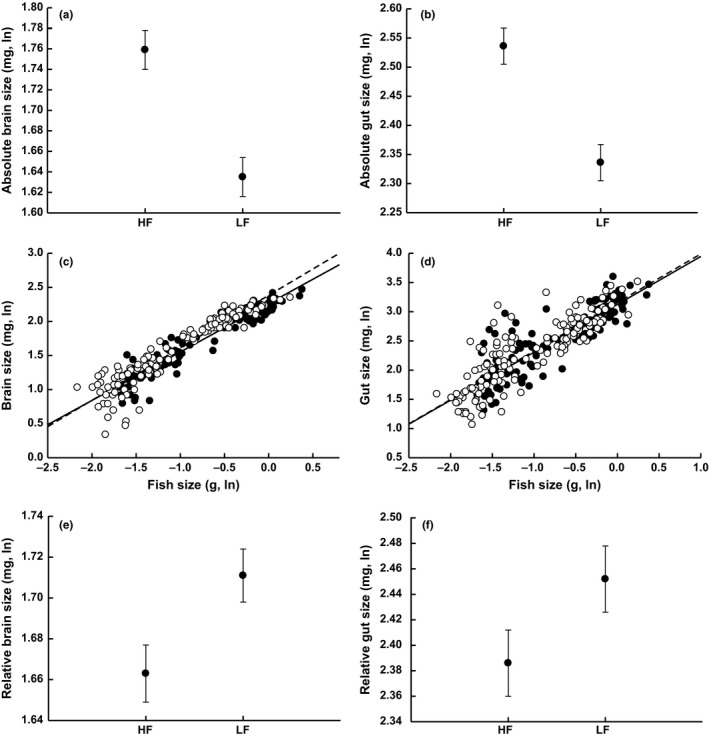
Influence of food treatments on brain and gut size. (a) absolute brain size, (b) absolute gut size, (c) fish size versus brain size, (d) fish size versus gut size, (e) relative brain size, (f) relative gut size. HF, high food; LF, low food. Panels c, d: closed circles (solid regression line)—high food; open circles (dashed regression line)—low food. We observed significant differences between the food treatments for absolute and relative brain size and absolute gut size. Differences in relative gut size were marginally nonsignificant (*p* < .1). The data points for relative brain and gut size reflect the estimated marginal means at the mean of the covariate. Error = ±1 *SE*

We observed a significant effect of food level on absolute and relative brain size (Table 1; Figure 3). Absolute brain size was 8% larger in fish fed high‐food when compared with the low‐food treatments (Figure 3). This trend was reversed for relative brain size; *Rivulus* fed a low‐food level exhibited a relative brain size that was 3% larger than the high‐food treatments. Absolute gut size differed significantly between the food treatments (Table 1; Figure 3); absolute gut size was 7% larger in high versus low‐food treatments. The effects of food level on relative gut size were marginally nonsignificant (*p* < .1) (Table 1; Figure 3). Relative gut size was 3% larger in the low‐food treatment versus the high‐food level.

### 3.3 Sex effects

**Figure 4:**
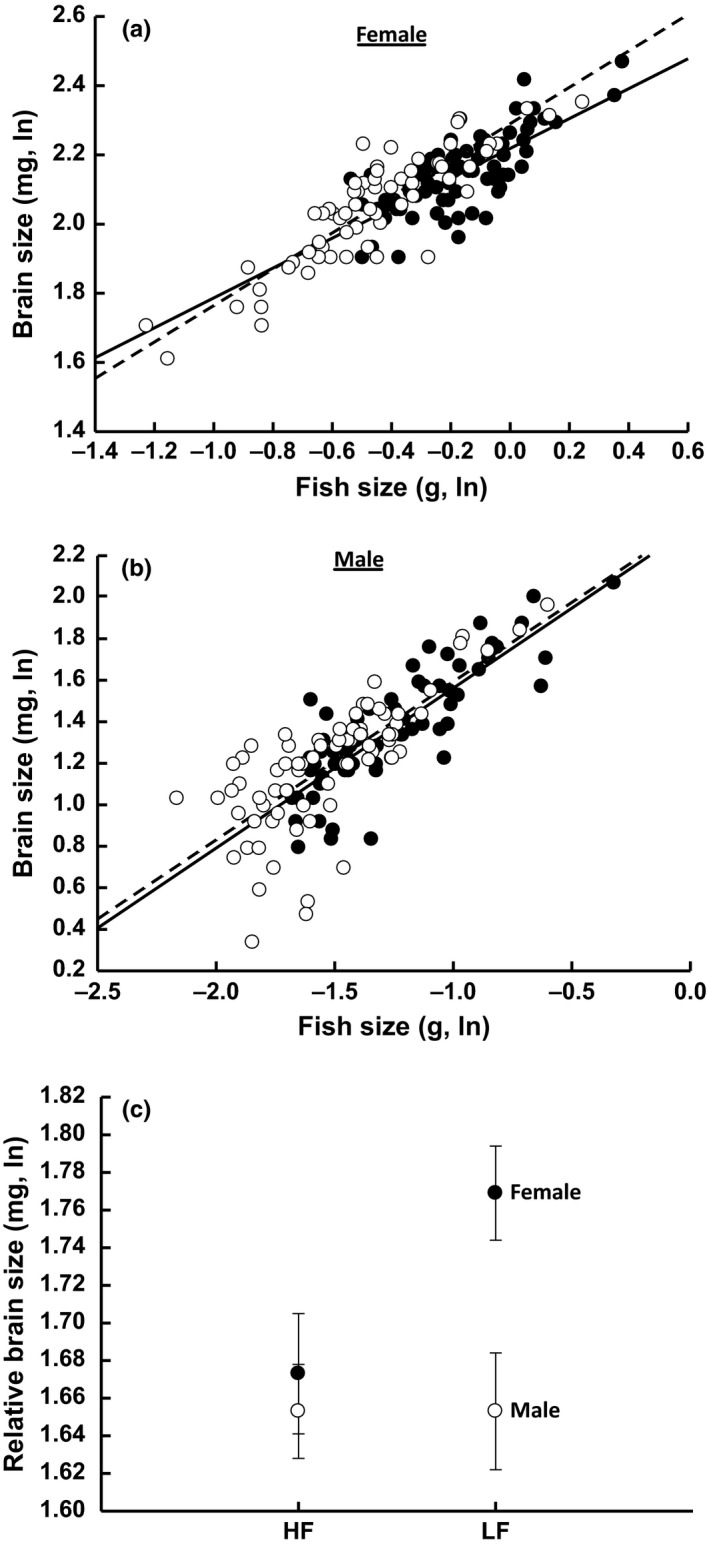
Sex‐specific responses in relative brain size as a function of food level. (a) female fish size versus brain size, (b) male fish size versus brain size, (c) sex by food interaction. Panels a, b: closed circles (solid regression) = high food, open circles (dashed regression)—low food. HF—high food, LF—low food. General linear models revealed a significant (*p* < .05) sex × food interaction for relative brain size. Error = ±1 *SE*

Absolute brain and gut size differed between the sexes (Table 1). The absolute brain and gut sizes were 63% and 45% larger in females than males (average ln absolute brain size (mg) ± 1 *SE*: females = 2.11 ± 0.019, males = 1.29 ± 0.019; average ln absolute gut size (mg) ± 1 *SE*: females = 2.88 ± 0.032, males = 1.99 ± 0.031). Patterns of divergence between the sexes for relative brain size depended upon controlled food levels in the laboratory as we observed a significant (*p* < .05) “food × sex” interaction (Table 1; Figure 4). The brain size of males differed little between high and low‐food levels (Figure 4). Conversely, females exhibited brains that were 5% larger under low versus high‐food levels (Figure 4).

## 4 Discussion

Our results show that increased rates of juvenile predation and correlated increases in resources in fish communities with guppies are not associated with consistent divergence in adult brain size of *Rivulus* (Table 1; Figures 1 and 3). Overall, the relative brain size between locations where *Rivulus* are (RG) and are not (RO) exposed to gape‐limited predation by guppies was nearly identical (Figure 1). Small differences (1%–3%) in relative brain size were observed between RG and RO sites, but these differences varied across rivers (i.e., significant population × river interaction) (Figure 2). It was recently shown that increased predation by large piscivores drives the evolution of substantially smaller brains in male (but not female) *Rivulus* (Walsh et al., 2016). Then: What explains the lack of consistent divergence in brain size between communities with and without guppies?

In our recent study, Walsh et al. (2016) tested the influence of predators on brain size evolution by comparing the brain size of *Rivulus* between sites that differ in the presence and absence of several species of large piscivorous fish (Walsh & Reznick, 2008, 2009). As described previously, *Rivulus* from sites with predators have evolved a smaller brain size (in males only). This is important because there are also known differences in prey behavior in this system (Fraser et al., 2001; Gilliam & Fraser, 2001). In sites with large predators, species such as *Crenicichla alta* and *Hoplias malabaricus* are capable of consuming all size classes of *Rivulus*. This increased predation is, in turn, associated with shifts in adult risk‐taking behavior; *Rivulus* are bolder in sites with large predators when compared with sites in which *Rivulus* are alone (Fraser et al., 2001; Gilliam & Fraser, 2001). We hypothesize that this covariation between behavior and brain size is due to the known differences in predator‐induced mortality (Fraser et al., 1999; Walsh et al., 2016).

The primary difference between the current study and previously completed work on *Rivulus* brain size evolution (see Walsh et al., 2016) is the nature of the predator community. In RG sites, juvenile *Rivulus* are subjected to predation by guppies (Fraser & Lamphere, 2013; Furness & Reznick, 2014), but quickly outgrow the gape of guppies. *Rivulus* also attain a much larger asymptotic size than guppies; adult guppies will grow up to 32 mm in total length (Rodd & Reznick, 1997), while *Rivulus* can attain a size of ~100 mm as adults (Walsh & Reznick, 2009). Life‐history theory and our subsequent empirical work clearly show that mortality targeted at immature age classes can shape adult life‐history traits (see Reznick & Endler, 1982; Stearns, 1992; Wellborn, 1994; Sparkes, 1996a; but also see Gadgil & Bossert, 1970; Law, 1979; Charlesworth, 1980; Fitzpatrick, Torres‐Dowdall, Reznick, Ghalambor, & Funk, 2014). In contrast to sites with large predators (Fraser et al., 2001; Gilliam & Fraser, 2001), guppies do not appear to alter the behavior of adult *Rivulus*. *Rivulus* in RG and RO sites are frequently observed in open water, rather than the stream margins when large predators are present (MR Walsh 2016, personal observation). As a result, we hypothesize that the lack of an association between juvenile mortality and selection on brain size is due to weak patterns of divergent selection on *Rivulus* learning and behavior between RG and RO sites. An alternative perspective is that cognitive demands differ when predation occurs throughout the lifetime in an organism versus when it is limited to a short duration early on in life. Regardless, these hypotheses require experimental testing.

It is important to note that we did detect small differences in brain size between RG and RO sites, but the direction of the differences varied across rivers (i.e., significant population × river interaction) (Table 1; Figures 1 and 2). For example, *Rivulus* from sites with guppies exhibited a relative brain size that was 3% larger than sites where *Rivulus* are alone in the Aripo river, but such trends were reversed in the Guanapo River (Figure 2). These small but variable responses further illustrate the lack of connection between the presence of guppies and evolutionary shifts in brain size. The cause of these variable differences in brain size between RG and RO sites across rivers is unclear. This is, in part, because we showed previously that these rivers and communities do not differ in the size of the physical habitat or in abiotic variables such as dissolved oxygen, salinity, or water temperature (Walsh & Reznick, 2009). However, it is certainly plausible that our focal streams could differ in other features that we have yet to account for (i.e., habitat complexity, flow regimes) and that may influence brain size divergence.

### 4.1 Brain size plasticity and resource availability

Our food treatments were designed to mimic natural variation in resource availability in the wild; *Rivulus* experience higher food availability in RG sites because predation by guppies is associated with declines in the abundances of *Rivulus* and, in turn, increased food for survivors (Fraser & Lamphere, 2013; Walsh et al., 2011). As expected, *Rivulus* attained a larger body size when reared on high versus low food (Figure 3). In turn, absolute brain size was significantly larger on high‐food levels when compared with low‐food levels (Table 1; Figure 3). However, such trends were reversed for relative brain size; *Rivulus* exhibited a larger relative brain size in the low‐food versus high‐food treatments (Table 1; Figure 3). These observed increases in brain size when food was reduced are largely due to a stronger response in females than males (Figure 4). One potential explanation for the differences in relative brain size between food treatments is that they are adaptive. For instance, increased brain size is broadly associated with higher levels of intelligence, problem‐solving abilities, and cognition across species (Benson‐Amram et al., 2016). It is thus plausible that larger brains are favored when resources are scarce because larger brains may improve foraging capabilities and ultimately fitness. Such plasticity may be adaptive as declines in foraging may foreshadow declining conditions. The divergent responses to reduced food between males and females also suggest that selection on brain size is perhaps stronger in females than males as efficient energy acquisition is likely to be especially important to maintaining high reproductive efforts.

An alternative explanation is that brain size is more canalized than body size, and thus, the differences in relative brain size between food treatments (or divergent responses to reduced food between males and females) are simply a byproduct of increased sensitivity of body size to resources. Regressions between body size and brain size revealed a positive relationship for high‐ and low‐food levels (Figure 3c), but the slope of this trend was higher for low‐food levels. Similarly, females exhibited a larger relative brain size when fed a low‐food rather than high‐food diet because brain size increased more rapidly as a function of body size in the fish fed a low‐food level versus high‐food level (Figure 4). These same regressions were nearly identical for males between the two food treatments (Figure 4). These regressions also indicate that the significant differences in relative brain size between food levels and the significant “sex × food” interaction are not likely due to increased canalization of brain size versus body size (see Fitzpatrick et al., 2012). The food treatments include fish that exhibit a similar range of variation in size, and the overall differences in body size between high‐ and low‐food levels for males and females are nearly identical (average ln male body size (g): HF = −1.28, LF = −1.52; average ln female body size (g): HF = −0.18, LF = −0.42). Our results instead foreshadow an adaptive connection between resource availability and brain–body size allometry, but such a hypothesis requires further testing.

### 4.2 Gut size variation

Similar to the patterns of brain size plasticity, declines in food were associated with the production of larger guts (Figure 3). This connection between gut size plasticity and food parallels much previous research (Benavides, Cancino, & Ojeda, 1994; Jackson, 1992; Korn, 1992; Olsson, Quevedo, Colson, & Svanbäck, 2007; Piersma & Lindstrom, 1997; Relyea & Auld, 2004; Siems & Sikes, 1998; Starck, 1996; Sullam et al., 2015; Wagner, McIntyre, Buels, Gilbert, & Michel, 2009). Optimal digestion theory predicts that low‐food quantities (or low‐quality food) should favor longer digestive tracts because this increase in gut size allows for an increase in resource absorbance efficiency (Kotrschal, Corral‐Lopez, Szidat, & Kolm, 2015c; Kotrschal, Szidat, & Taborsky, 2014; Relyea & Auld, 2004; Savory & Gentle, 1976; Yang & Joern, 1994).

Overall, females produced larger guts than males (Table 1). Similar to the observed differences in brain size under low‐food conditions between males and females, these differences in gut size between the sexes are potentially explained by differences in energy budgets, specifically that females allocate more energy to reproduction and may need to ensure maximal conversion of resources into reproductive tissue. Such a notion is supported by sex‐specific differences in gut size in other taxa (Hudry, Khadayate, & Miguel‐Aliaga, 2016; Reiff et al., 2015). For example, the organ responsible for absorption of nutrients in *Drosophila melanogaster*, the midgut, is not only longer in females when compared to male fruit flies, but increases in length following mating (Reiff et al., 2015).

### 4.3 Ecological drivers of brain size evolution

Interest in the relationship between ecological forces and selection on brain size is growing rapidly. Research has shown that ecological variables, such as social environment (Connor, 2007; Kotrschal, Rogell, Maklakov, & Kolm, 2012a; Shultz & Dunbar, 2006b), diet (Allen & Kay, 2012; Shultz & Dunbar, 2006a), habitat (Crispo & Chapman, 2010; Gonda et al., 2009b; Kotrschal, Sundstrom, Brelin, Devlin, & Kolm, 2012b), and predators (Gonda et al., 2009a,b, 2011; Walsh et al., 2016), play an important role in brain size plasticity and brain size evolution (see also Gonda, Herczeg, & Merila, 2013). These latter studies exploring the connection between predators and selection on brain size have largely compared populations where adults are susceptible to predators (Gonda et al., 2009a,b, 2011; Walsh et al., 2016). For example, Gonda et al. (2009a,b, 2011) compared patterns of brain size variation in sticklebacks from divergent aquatic habitats. Sticklebacks in environmentally complex marine habitats experience high levels of predation and lower densities, while simple pond environments lack predators (Gonda et al., 2011). Results from this work showed that wild‐caught sticklebacks from marine habitats exhibited smaller brains than fish from ponds (Gonda et al., 2009a). Such trends largely parallel those observed in *Rivulus* between sites with and without large predators (for males only) (Walsh et al., 2016) although the brain size differences in sticklebacks were not maintained in common garden‐reared fish (Gonda et al., 2011). Conversely, work on captive populations of guppies showed that selection for a larger brain is associated with enhanced survival in risky habitats (Kotrschal et al., 2015a). This growing body of work clearly provides a connection between predators and brain size, although the contradictory nature of these results currently limits our understanding of the manner in which predatory selection acts on brain size.

Our study advances work exploring connections between predation and brain size because it examines populations that are exposed to predation during a brief, discrete interval of time. Despite extensive research demonstrating a link between mortality targeted at immature age classes and resultant selection on a suite of adult characteristics (Hutchings, 1993; Reznick & Endler, 1982; Sparkes, 1996a; Stearns, 1992; Walsh & Reznick, 2010, 2011; Wellborn, 1994), we did not find an association between increased juvenile predation and evolutionary shifts in brain size. One potential implication of our results is that they provide a window into the time period in which selection does or does not act on brain size and indicates that variation in adult mortality may be a strong predictor of brain size evolution in nature. Direct tests of the influence of increased juvenile or adult mortality on the evolution of brain size are now needed.

## 5 Conclusions

Here, we tested the influence of early‐life mortality on brain size evolution. Despite a growing body of work illustrating a connection between predator‐induced mortality and the evolution of vertebrate brain size (van der Bijl et al., 2015; Burns & Rodd, 2008; Edmunds et al., 2016; Kotrschal et al., 2012b, 2015b; Shultz & Dunbar, 2006a; Walsh et al., 2016), we found that increased rates of juvenile mortality are not associated with evolutionary shifts in adult brain size. In contrast to much work illustrating a connection between juvenile mortality and evolutionary shifts in adult characteristics (Reznick & Endler, 1982; Sparkes, 1996a; Wellborn, 1994), one potential implication of our results is that mortality experienced early in life may not alter selection on adult brain size.

## Conflict of interests

None declared.
