# Supplementary material for: Increased juvenile predation is not associated with evolved differences in adult brain size in Trinidadian killifish (Rivulus hartii)
Source: Ecol Evol. 2017 Jan 12;7(3):884–94. doi: 10.1002/ece3.2668 (PMC5288286; doi:10.1002/ece3.2668)
Supplement: Supplementary file 1 [file ECE3-7-884-s001.docx]

**SUPPORTING INFORMATION**

**Table S1.** Average relative gut size for ‘river x population’ interaction. Marginal means of natural log transformed gut size data are reported as relative gut size (mg, ln) for *Rivulus-*only (RO) and *Rivulus-*guppy (RG) populations in the Aripo, Guanapo, and Quare rivers.

|  |  | **Relative gut size (mg, ln)** | **Standard error** |
| --- | --- | --- | --- |
| **River** | **Population** |  |  |
| Aripo | RO | 2.391 | 0.038 |
|  | RG | 2.369 | 0.037 |
|  |  |  |  |
| Guanapo | RO | 2.439 | 0.029 |
|  | RG | 2.418 | 0.03 |
|  |  |  |  |
| Quare | RO | 2.456 | 0.036 |
|  | RG | 2.435 | 0.036 |
